# Bacteriocin Mining in *Lactiplantibacillus pentosus* PCZ4 with Broad-Spectrum Antibacterial Activity and Its Biopreservative Effects on Snakehead Fish

**DOI:** 10.3390/foods13233863

**Published:** 2024-11-29

**Authors:** Hechao Du, Siyu Li, Hongliang Yao, Nannan Wang, Ruiqiu Zhao, Fanqiang Meng

**Affiliations:** 1 College of Animal Science and Food Engineering, Jinling Institute of Technology, 130 Xiaozhuang Central Village, Nanjing 210046, China; lisis@chinacoal-ins.com (S.L.); dlyaohongliang@jit.edu.cn (H.Y.); wangnn@jit.edu.cn (N.W.); zhaoruiqiu9210@jit.edu.cn (R.Z.); 2 College of Veterinary Medicine, Nanjing Agricultural University, 1 Weigang, Nanjing 210095, China; 3 College of Food Science and Technology, Nanjing Agricultural University, 1 Weigang, Nanjing 210095, China; mfq@njau.edu.cn

**Keywords:** lactic acid bacteria, whole genome sequencing, bacteriocin mining, antibacterial, fish preservation

## Abstract

Some lactic acid bacteria (LAB) produce antibacterial substances such as bacteriocins, making them promising candidates for food preservation. In our study, *Lactiplantibacillus pentosus* PCZ4—a strain with broad-spectrum antibacterial activity—was isolated from traditional fermented kimchi in Sichuan. Whole-genome sequencing of PCZ4 revealed one chromosome and three plasmids. Through BAGEL4 mining, classes IIa and IIb bacteriocin plantaricin S were identified. Additionally, two new antibacterial peptides, Bac1109 and Bac2485, were predicted from scratch by limiting open reading frames. Furthermore, during refrigerated storage of snakehead fish, PCZ4 crude extract reduced the total bacterial count, slowed the increase in TVB-N and pH values, improved the sensory quality of the snakehead, and extended its shelf life by 2 days. Meanwhile, PCZ4 effectively inhibited the growth of artificially contaminated *Aeromonas hydrophila* in snakehead fish. These findings indicate that *Lp. pentosus* PCZ4 can produce multiple antibacterial substances with strong potential for food preservation applications.

## 1. Introduction

Amid growing consumer concerns regarding food safety, chemical preservatives are being scrutinized due to their potential health risks. This has led to a surge in the demand for natural biological preservatives. Lactic acid bacteria (LAB)—particularly those from traditional fermentation processes—are promising candidates owing to their inhibitory activities, and they are generally recognized as safe. LAB can produce various inhibitory substances, including organic acids, hydrogen peroxide, diacetyl, and most notably, bacteriocins. Bacteriocins are ribosomally synthesized peptides that are generally safe for human consumption and effective against foodborne pathogens and spoilage microorganisms, making them a focal point for natural food preservation [1]. Bacteriocins have diverse structures, resulting in several categories [2,3]. The most commonly used classification system groups LAB bacteriocins into three main classes [4,5]: low molecular weight (<10 kDa), heat-stable, post-translationally modified bacteriocins (class I); unmodified peptides (class II); and thermolabile bacteriocins with a molecular weight > 10 kDa (class III, e.g., enterolysin A) [6]. Class I bacteriocins are further subdivided into classes Ia and Ib, based on whether or not their structure is cyclic. Class II is comprised of four subclasses: class IIa (pediocin-like, e.g., pediocin PA-1), class IIb (two-peptide, e.g., plantaricin EF), class IIc (cyclic, e.g., enterocin AS-48), and class IId (non-pediocin-like single-peptide) [7,8].

Bacteriocins can be used as biopreservatives in the following three ways: (i) as pure bacteriocin preparations, (ii) as bacteriocin-containing fermentates, or (iii) as bacteriocin-producing cultures [9]. The most extensively studied bacteriocin, nisin A—produced by *Lactococcus lactis*—is the only commercially produced bacteriocin approved by regulatory agencies as a food additive [10]. Commercially available products such as Nisaplin (2.5% nisin) can be used to preserve meat, dairy products, canned foods, and fruit juice beverages. Furthermore, bacteriocin-containing food-grade fermentates are commercially available and widely used in the food industry, including the Food and Drug Administration-approved Micro-GARD^TM^, from Danisco, and ALTA 2431, from Quest International. Both Micro-GARD^TM^ and ALTA 2431 contain PA-1 produced by *Pediococcus acidilactici* [5]. Pediocin PA-1 exhibits excellent effects against *Listeria monocytogenes* and inhibitory activity against other Gram-positive bacteria, such as *Staphylococcus aureus* and *Bacillus subtilis* [11]. Bacteriocin-producing cultures—used as starter or adjunct cultures—serve a dual function by enhancing flavor and food safety by simultaneously providing fermentation and preservation. Examples include the Bactoferm^TM^ range, which contains pediocin- and sakacin-producing strains used to produce fermented sausages and dry-cured meat [12]. Recently discovered novel bacteriocins, such as bacteriocin ZFM216 [13], BM1029 [14], enterocin 12a [15], plantaricin EmF [16], and plantaricin KMY15 [17], demonstrate strong potential as food preservatives by reducing the pathogen populations involved in food preservation.

To date, numerous bacteriocins have been isolated. However, only nisin and pediocin PA-1 have gained approval for food processing applications. These bacteriocins exhibit limitations such as a reduced capacity to exert antibacterial effects under neutral or alkaline pH conditions. Furthermore, their efficacy is predominantly observed in Gram-positive bacteria. Consequently, this reveals the urgent need for novel broad-spectrum bacteriocins capable of addressing the risks posed by pathogenic and spoilage bacteria in the food supply. Genome mining—also known as in silico analysis—has improved the discovery of bacteriocins by identifying biosynthetic gene clusters and pathways within bacterial genomes. Bacteriocin mining tools such as BAGEL4 have been instrumental in these efforts [18]. For instance, a study by Teber et al. [19] indicated that 60.78% of the 51 marine *Bacillota* genomes examined were potential bacteriocin producers, identifying 77 bacteriocin gene clusters using BAGEL4. BAGEL is also used to mine and classify bacteriocins based on their similarity to identified bacteriocins, the presence of motifs, and associated features. Using the BAGEL4 web server, two bacteriocin clusters, namely sactipeptide and plantaricin J, were identified in the *Lactiplantibacillus plantarum* DJF10 strain, indicating its enhanced antimicrobial potential [20]. Bacteriocin mining demonstrated the presence of a complete bacteriocin production gene cluster in *Lp. plantarum* WLPL21, including the *plnJKR*, *plnPQAB*, *plnEFI*, and *plnSUVWY* genes [21]. Since the inception of BAGEL, several other bacteriocin mining servers—such as antiSMASH and RIPPMiner—have been developed, further expanding the scope of in silico bacteriocin discovery.

Aquatic products—renowned for their nutritional content—are inherently susceptible to spoilage and overall deterioration, primarily due to the rapid growth of pathogenic and spoilage bacteria. The predominant bacteria include pathogens such as *Vibrio parahaemolyticus* and *Shigella* spp., as well as spoilage and potentially pathogenic bacteria such as *Aeromonas hydrophila* and *Pseudomonas* spp., all of which are Gram-negative. Therefore, in this study, we aimed to identify LAB that exhibit broad-spectrum antibacterial activity and mine their antibacterial genes through whole-genome sequencing (WGS), with the aim of enhancing the use of LAB and their antibacterial products in aquatic products.

## 2. Materials and Methods

### 2.1. Screening and Characteristics of the PCZ4 Strain

#### 2.1.1. Isolation and Purification of LAB

Traditional fermented kimchi samples were obtained from the Guizhou, Sichuan, and Liaoning provinces in China. Each sample (25.0 g) was homogenized and serially diluted 10-fold with sterile saline (0.85% NaCl, *w*/*v*). Subsequently, 200 μL of each dilution solution was plated on de Man, Rogosa, and Sharpe (MRS) agar plates (Hopebio, Qingdao, China). After incubation at 37 °C for 48 h, colonies exhibiting LAB-like features—smooth, round protrusions, milky white color, and neat edges—were selected. The isolates were then purified and stored in MRS broth containing 25% (*v*/*v*) glycerol at −20 °C.

#### 2.1.2. Screening of LAB with Antibacterial Activity

The antibacterial activity of the LAB strains was determined using the agar diffusion method [22]. Each LAB isolate was incubated in MRS broth at 37 °C for 48 h. The culture was centrifuged at 8000× *g* for 10 min at 4 °C before being filtered using a 0.22 μm pore-size filter to obtain the cell-free supernatant (CFS). The pH of the CFS was adjusted to 5.5 with NaOH. *Escherichia coli* CMCC 44102 and *Staphylococcus aureus* subsp. aureus ATCC 6538 were used as indicator bacteria. A 1 mL logarithmic-phase culture (approximately 10^8^ cells) was added to 100 mL of boiled Luria–Bertani (LB) agar (1.5%, *w*/*v*), which was cooled to 50 °C. The LB agar containing the indicator bacteria was poured into a 10 cm diameter plate, and 5 mm diameter wells were punched. Subsequently, 50 μL of each CFS was added to the wells, and the plates were incubated overnight at 37 °C. The antibacterial activity was evaluated based on the diameter of the inhibition zone.

The antibacterial spectrum of the LAB strains was determined using the agar diffusion method. The indicator bacteria included *Pseudomonas aeruginosa*, *Aeromonas aeruginosa*, *Bacillus subtilis*, *Salmonella*, *E. coli*, and *S. aureus*. Among these, *E. coli* CMC 44102, *B. subtilis* ATCC 6633, *S. aureus* ATCC BAA-1717, and *S. aureus* subsp. ATCC 6538 were purchased. The other strains were isolated from samples of diseased pigeons, crucian carp, and farmed chickens. They were identified through morphological, biochemical, and 16S rRNA PCR analyses. All samples were frozen in our laboratory.

#### 2.1.3. Tolerance to Gastric Juice

LAB culture (10 mL) in the logarithmic phase was centrifuged at 8000× *g* for 10 min. The bacterial precipitate was washed twice with phosphate-buffered saline (PBS) (10 mM, pH 7.4) and resuspended in 1 mL of PBS. The simulated gastric juice was prepared following the previous method, with some modifications [23]. The pH was adjusted to 3.0 by adding 5 M HCl. After autoclaving, 1.0 g of pepsin was added and thoroughly mixed. The prepared artificial gastric juice and bacterial suspension were incubated at a volume ratio of 9:1 at 37 °C for 3 h. The live bacterial counts were determined at 0 and 3 h. Tolerance to acidic conditions was measured based on the survival rate, which was calculated using Equation (1).

C = (m2/m1) × 100%(1)

C—survival rate, %.

m2—live bacterial count at 0 h, CFU/mL.

m1—live bacterial count at 3 h, CFU/mL.

#### 2.1.4. Tolerance to Bile Salts

LAB culture (2 mL) in the logarithmic phase was incubated into MRS broth containing different bile salt concentrations (*w*/*v*) (0.0%, 0.2%, 0.3%) and incubated at 37 °C for 4 h. The OD_600_ of each bacterial suspension was measured at 0 and 4 h. Tolerance to bile salts was measured based on the growth rate [24].

The growth rate of the LAB strains was calculated using Equation (2).

C = (A3 − A1)/(A2 − A0)(2)

C—growth rate, %.

A0—OD_600_ value of 0.00% bile salt culture medium at 0 h.

A1—OD_600_ value of 0.00% bile salt culture medium at 4 h.

A2—OD_600_ values of culture media with different bile salt concentrations at 0 h.

A3—OD_600_ values of different bile salt concentration culture media at 4 h.

#### 2.1.5. Growth and Antibacterial Kinetics Analyses

The PCZ4 culture (3 mL) was incubated in 100 mL of MRS broth at 37 °C for 48 h. The OD_600_ was measured every 2 h using a Nano-600 ultra-trace nucleic acid and protein analyzer (Shanghai Jinpeng Analytical Instrument Co., Ltd., Shanghai, China). Simultaneously, 3 mL of fermentation broth was pipetted at each time point to prepare CFS, and the pH value was measured using a BPP-7800 pH meter (BELL Analytical Instruments Co., Ltd., Dalian, China). The inhibition zones against *E. coli* CMC 44102 and *S. aureus* subsp. ATCC 6538 were evaluated as described in Section 2.1.2.

### 2.2. Genome Sequencing and Analysis

#### 2.2.1. Culture and DNA Extraction of Bacterial Strains

The PCZ4 culture was transferred to MRS broth at a 3% (*v*/*v*) ratio and incubated at 37 °C for 12 h. Bacterial cells were collected via centrifuge (8000× *g* for 10 min at 4 °C). After washing twice with sterile PBS, the DNA was extracted using a DNA extraction kit (Beijing Solaibao Technology Co., Ltd., Beijing, China), according to the manufacturer’s instructions. The purity, concentration, and integrity of the extracted genomic DNA were determined using a Nano-600 ultra-micro nucleic acid and protein analyzer and agarose gel electrophoresis. The qualified sample (OD_260/280_ = 1.8–2.0, DNA concentration ≥ 50 ng/μL, total DNA amount ≥ 100 μg) was sent to Shanghai Meiji Biomedical Technology Co., Ltd. (Shanghai, China) for library construction and sequencing.

#### 2.2.2. Genome Sequencing, Assembly, and Annotation

Genome sequencing was conducted by combining the second Illumina MiSeq and third-generation PacBio platforms (Pacific Biosciences, Menlo Park, CA, USA). The complete genome sequence was generated by de novo assembly using Unicycler v0.4.8, and a genomic circular map was created using Circos software [25]. A phylogenetic tree of strain PCZ4 was constructed based on 31 housekeeping genes. The neighbor-joining method was employed to construct the phylogenetic tree using MEGA 6.0 software.

The coding sequences (CDS) within the genome were predicted using Glimmer, GeneMarkS, and Prodigal software. Functional annotation was conducted via a BLAST search of different databases, including Gene Ontology (GO), Clusters of Orthologous Groups of Proteins (COG), and the Kyoto Encyclopedia of Genes and Genomes (KEGG). The secondary metabolite synthesis gene cluster was analyzed via the online software antiSMASH.

#### 2.2.3. Identification and Analysis of Novel Bacteriocins

Two approaches were employed to minimize bacteriocins in the genome: (1) comparison with and searching within databases and (2) prediction from scratch by limiting open reading frames (ORFs). Bacteriocin gene clusters were mined using BAGEL4 RiPP miners (accessed on 9 June 2024) [18]. Furthermore, new bacteriocins were identified using the second method. First, EMBOSS software was used to filter ORFs within 60–200 bp. SignalP-6.0 was employed for transmembrane structural analysis to identify ORFs containing signal peptides. CAMPR4 and AntiBP2 software were used to screen the ORFs for antibacterial activity. To determine whether the predicted bacteriocins were known or novel, the ORFs were compared using the NCBI and APD3 databases. Finally, the physicochemical properties and structures of the predicted bacteriocins were analyzed using the Expasy, NPSA, and SWISS-MODEL servers.

### 2.3. Bio-Preservation of PCZ4 Crude Extract in Snakehead Fish

#### 2.3.1. Preparation of an Antibacterial Crude Extract

PCZ4 bacteriocin was extracted from 100 mL of CFS using an ethyl acetate extraction method at a 1:1 volume ratio, which was obtained via preliminary experimental optimization. After vortexing for 30 min, the mixed solution was incubated for 1 h. The upper organic layer was then rotary evaporated at 45 °C for 1 h until no residual ethyl acetate remained. The solution was freeze-dried and redissolved in PBS for subsequent experiments.

#### 2.3.2. Determination of the Minimum Inhibitory Concentration

The minimum inhibitory concentration (MIC) of PCZ4 against *Aeromonas hydrophila* was determined using a 96-well plate [26]. The logarithmic-phase culture of *A. hydrophila* was adjusted to an OD_600_ of 0.4 and diluted 100-fold in LB broth. In a 96-well plate, 100 μL of PCZ4 crude extract was two-fold serial diluted with PB before mixing in 100 μL of *A. hydrophila* suspension. The plates were incubated at 37 °C for 24 h. The MIC was determined by measuring the OD_600_ in a microplate reader (BioTek Instruments, Winooski, VT, USA). The MIC was defined as the minimum concentration of bacteriocin in the nonbacterial growth well, and this concentration was used for the sample treatment.

#### 2.3.3. Preparation of the Snakehead Sample

Fresh snakeheads were purchased from a local market and transported to the laboratory in iceboxes. After slaughter, each snakehead was cut into four pieces weighing approximately 50.0 g. The samples were evenly divided into four groups (A1, A2, B1, and B2) and treated as follows: Group A1 was soaked in sterile water (control); Group A2 was soaked in 1 × MIC of PCZ4 crude extract; in group B1, the *A. hydrophila* suspension was added (final concentration of approximately 10^5^ CFU/g); and in group B2, after adding *A. hydrophila* suspension, the fish sample was soaked in 1 × MIC of the crude extract. Each soaking step lasted 10 min, followed by air drying at 25 °C until visually dry. In total, the snakehead samples were distributed across four groups, five time points, and three replicates. Each sample from the four groups was obtained on days 1, 3, 5, 7, 9, and 11 of storage for fish quality analysis.

#### 2.3.4. Microbiology Analysis

The mixture of fish sample (10.0 g) and saline solution (90 mL) was crushed using a homogenizer. The homogenate was then diluted 10-fold in saline solution. The diluted solution (100 μL) was plated onto Plate Count or Rimler–Shotts agar (Hopebio, Qingdao, China) to assess total viable counts and *A. hydrophila*, respectively. The bacterial counts were recorded as Log_10_ CFU/g of the fish sample.

#### 2.3.5. Determination of the Total Volatile Basic Nitrogen (TVB-N)

The TVB-N was determined using the semi-micro diffusion method following the National Food Safety Standard GB 5009.228-2016 (Beijing, China) [27]. The stirred sample (20.0 g) was added to 100.0 mL of distilled water and soaked for 30 min. After filtration, 10.0 mL of the filtrate and 5.0 mL of MgO (10.0 g/L) were mixed in a Kjeldahl distillation unit (1765, Nanjing Tenghui Experimental Equipment Co., Ltd., Nanjing, China). After distillation for 5 min, the released volatile basic nitrogen gas was directed into a receiving bottle containing 10 mL of boric acid solution (20 g/L) and five drops of an indicator solution (methyl red ethanol solution–bromocresol green ethanol solution = 1:5 (*v*/*v*)). The boric acid solution was then titrated with standard hydrochloric acid (0.0100 mol/L) to the endpoint. The T-VBN content was expressed as mg/100 g.

#### 2.3.6. pH Determination

A 10.0 g portion of each sample was homogenized with 100 mL of distilled water. After filtration, the pH of the resulting filtrate was determined using a pH meter.

#### 2.3.7. Surface Color Determination

The surface color was described using three coordinates: lightness (L*), redness (a*), and yellowness (b*). Color values were measured using an NR110 chromatic meter (Guangdong Threenh Technology Co., Ltd., Guangzhou, China). The color difference (ΔE) was calculated using the formula ΔE = (ΔL)^2^ + (Δa)^2^ + (Δb)^2^.

#### 2.3.8. Sensory Evaluation

The sensory evaluation team consisted of eight trained panelists. At the same time points, all experimental samples were randomly coded and distributed to each panelist for evaluation based on color, odor, elasticity, and stickiness. Each attribute was scored on a 10-point Likert scale. The highest total score was 40 points (10 × 4).

### 2.4. Data Processing

Each experiment was conducted in triplicate, and data are expressed as the mean ± standard deviation. The significance of the data was assessed using one-way analysis of variance (ANOVA), followed by Duncan’s multiple range test, using SPSS version 20.0. Graphs were generated using Origin 2021.

## 3. Results

### 3.1. Screening and Characteristics of LAB

#### 3.1.1. Antibacterial Activity

In total, 55 LAB-like morphology strains were isolated from fermented kimchi samples. Through initial antibacterial screening, ten strains, including PCZ4, PZ1, L1, P27, P23B, YC3B, HPZ2A, HPZ2B, HPZ2C, and YY16, exhibited significant antibacterial effects, with inhibition zones ≥ 10 mm. PCZ4, PZ1, HPZ2A, HPZ2B, and HPZ2C were isolated from the kimchi samples of Guizhou Province; P27 and P23B were isolated from the kimchi samples of Liaoning Province; and YC3B, YY16, and L1 were isolated from the kimchi samples of Sichuan Province. The antibacterial spectra of the ten LAB-like morphology strains are presented in Table 1. All ten LAB-like morphology strains were effective against both Gram-positive and Gram-negative indicator bacteria. Overall, strains PCZ4 and PZ1 showed the best antibacterial effect, and the difference between them against the same indicator bacteria was not significant (*p* > 0.05).

#### 3.1.2. Tolerance to pH and Bile Salts

The tolerance of the ten LAB-like morphology strains to simulated gastric juice and bile salts was evaluated (Table 2).

After 3 h of culturing in simulated gastric juice, the PCZ4, P27, P23B, YC3B, HPZ2A, and YY16 strains exhibited survival rates of > 100% (110.72%, 133.50%, 138.24%, 111.23%, 160.47%, and 144.00%, respectively), indicating robust acid resistance with viable counts > 10^9^ CFU/mL.

In MRS broth containing 0.2% bile salts, strains L1, PZ1, and PCZ4 showed significant tolerance, with growth rates of 75.8%, 66.8%, and 66.7%, respectively. When the bile salt concentration was increased to 0.3%, the growth rate of the salts declined. After 4 h of stress treatment, PZ1 exhibited outstanding performance with a growth rate of 65.5%, followed closely by PCZ4 at 63.6%, indicating that PZ1 and PCZ4 exhibit outstanding bile salt tolerance among the ten strains.

Based on these results, both the PCZ4 and PZ1 strains exhibited outstanding antibacterial activity and high tolerance to bile salts. However, the survival rate of PZ1 in simulated gastric juice was poor (48.33%). Thus, PCZ4 was selected as a candidate for further evaluation.

#### 3.1.3. Growth and Antibacterial Kinetics Curves

**Figure 1:**
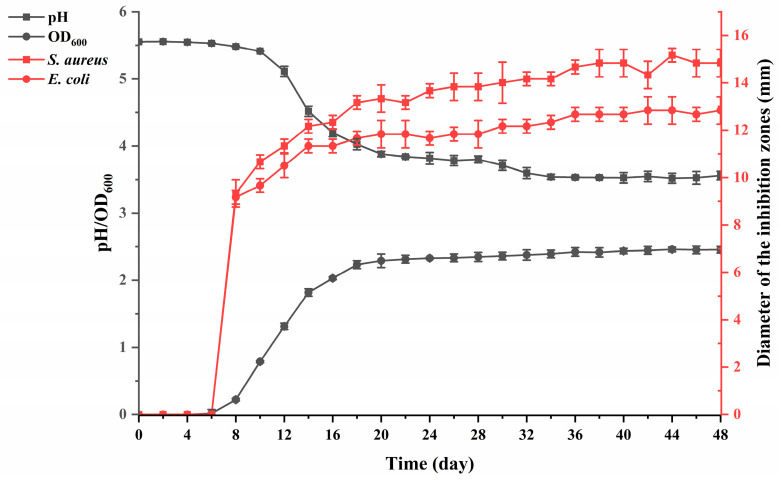
Growth and antibacterial dynamics of PCZ4.

Figure 1 shows that PCZ4 entered the logarithmic phase after 8 h of cultivation at 37 °C. After 20 h, PCZ4 entered the stationary phase at an OD600 of 2.20. Following this, no significant change in the OD_600_ value was observed. The initial pH was 5.5, which significantly decreased after 8 h. This indicates that as the strain grows into the logarithmic phase, the pH value begins to decrease significantly. After 34 h of cultivation, the pH stabilized at approximately 3.5. Similarly, strain PCZ4 began to show antibacterial activity against *E. coli* and *S. aureus* after 8 h of cultivation at 37 °C, indicating the rapid growth of PCZ4. The diameter of the inhibition zone then continued to increase and stabilized after 38 h.

### 3.2. WGS and Bioinformatic (In Silico) Analysis

#### 3.2.1. General Genomic Features of the *Lp. pentosus* PCZ4 Strain

**Figure 2:**
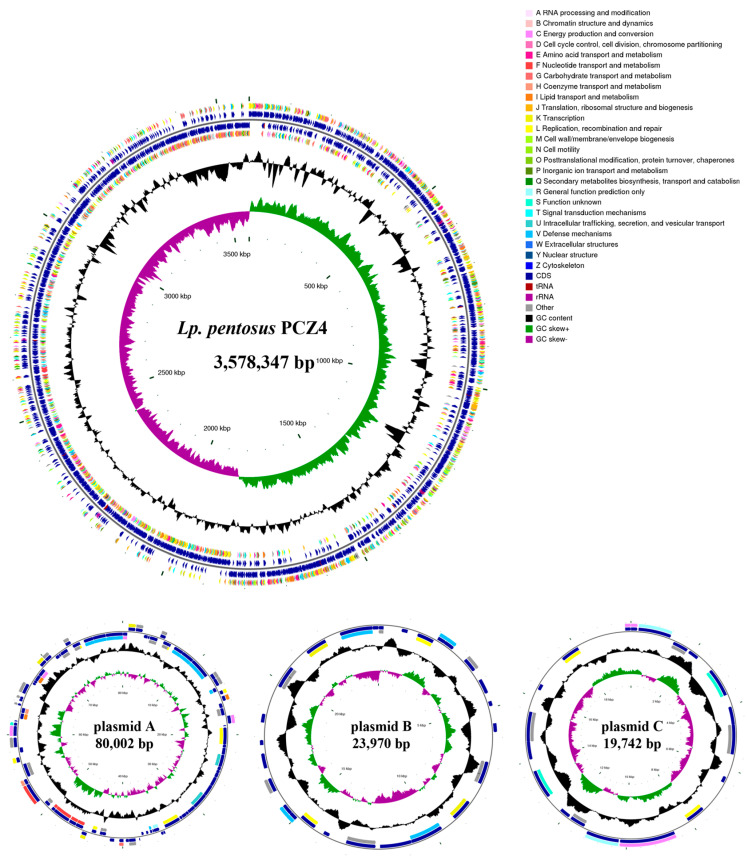
Genomic map of PCZ4 chromosomes and plasmids A, B, and C.

The *Lp. pentosus* PCZ4 genome was successfully assembled on the second Illumina and third-generation PacBio platform. The genome consisted of a single circular chromosome and three distinct circular plasmids. The mean chromosome length was 3,702,061 bp, with an average GC content of 46.19%. Plasmids A, B, and C had lengths of 80,002, 23,970, and 19,742 bp and GC contents of 41.73%, 37.38%, and 35.55%, respectively. A comprehensive genomic analysis revealed that *Lp. pentosus* PCZ4 contained 3283 coding genes (CDS) with a length of 2,986,296 bp, accounting for 80.67% of the genome sequence. The WGS data were deposited in the NCBI GenBank under the following accession numbers: chromosome (CP162863), plasmidA (CP162864), plasmidB (CP162865), and plasmidC (CP162866). The relevant genome features and distributions are represented in circular genome maps (Figure 2).

**Figure 3:**
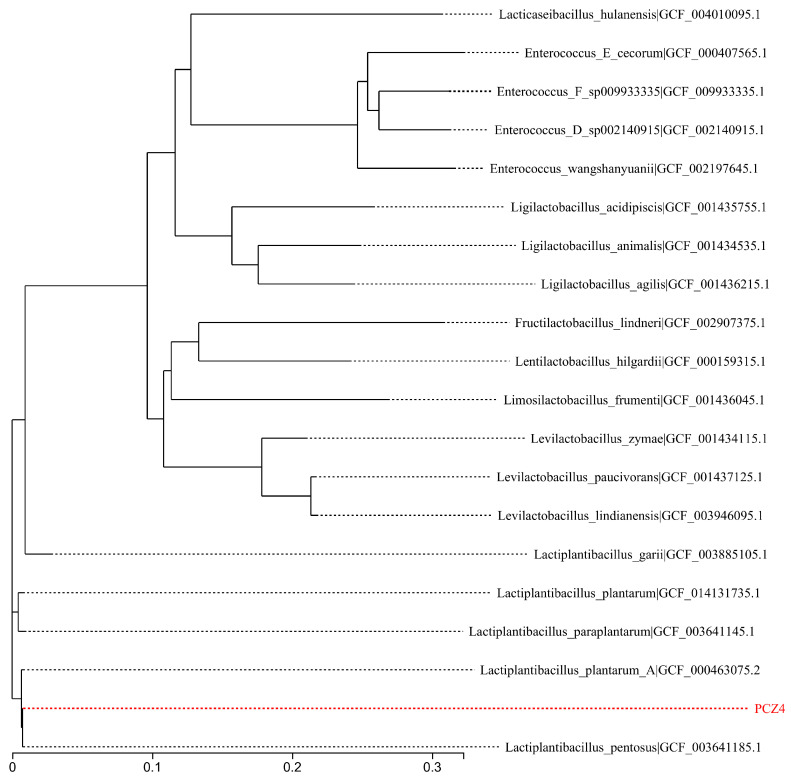
Phylogenetic tree of the PCZ4 strain.

To gain further insight into the phylogenetic position and evolutionary relationships of the clones, a phylogenetic tree was constructed based on the 31 housekeeping gene sequences of the 19 strains closest to the target strain at the genus level (Figure 3). The PCZ4 strain formed a cluster with *Lactobacillus pentosus* (previously known as *Lactobacillus pentosus*) and *Lp. plantarum*, both of which exhibit very close evolutionary relationships and are easily confused. Therefore, the average nucleotide identity was determined for the sake of rigor. The genome sequence of PCZ4 was 97.94% identical to the genome of *Lp. pentosus*, with 87.8% genome coverage. Therefore, PCZ4 was defined as *Lp. pentosus*, and named *Lp. pentosus* PCZ4.

Table 3 presents the general genome feature statistics of PCZ4, including genomic assembly, functional database annotations, and mobile genetic elements. Only one prophage was annotated, which can be considered an additional positive trait for probiotic candidates. Twelve CRISPRs were annotated, which revealed minimal antibiotic resistance.

#### 3.2.2. Genome Functional Annotation Analysis of PCZ4

**Figure 4:**
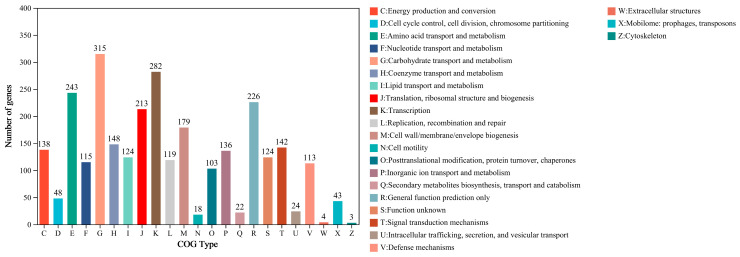
COG function annotation statistics for *Lp. plantarum* PCZ4.

The functional annotation of PCZ4 was conducted by comparing it with six other databases (NR, Swiss-Prot, Pfam, COG, GO, and KEGG) (Table 3). Among the 3283 genes, 2575 (78.43%), 2031 (61.84%), and 1767 (53.82%) genes were annotated using the commonly used COG, GO, and KEGG databases, respectively. The COG annotation (Figure 4) revealed that most annotated genes were involved in metabolism (1241 genes, 48.19%), followed by cellular processes and signaling (677 genes, 26.29%). Among them, 315 CDS were associated with carbohydrate transport and metabolism. This finding highlights the high activity of cells in energy production and consumption and the demand for complexity and diversity in the carbohydrate metabolic pathways. In addition, 282 CDS were associated with transcription, highlighting the role of cells in regulating gene expression and protein synthesis.

**Figure 5:**
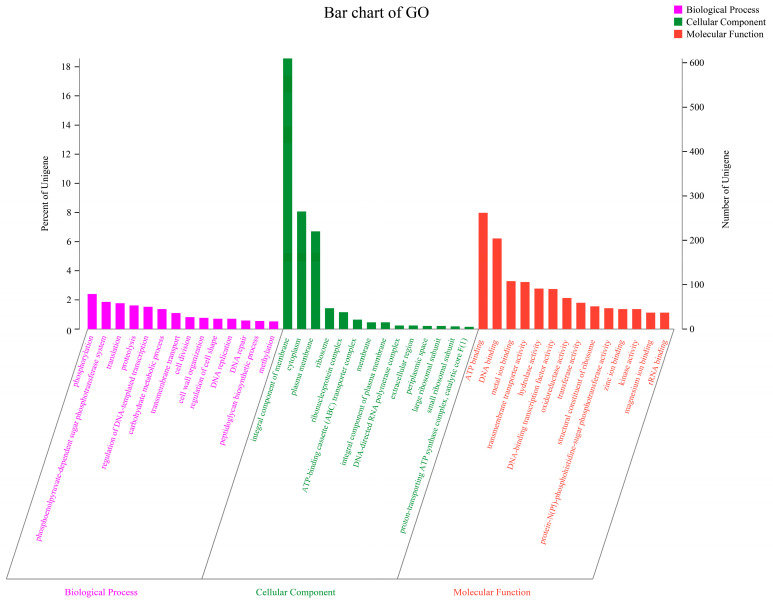
GO function annotation statistics for *Lp. plantarum* PCZ4.

GO provided three classifications: cellular component (1045 genes), molecular function (1625 genes), and biological process (1011 genes). Among them, significantly enriched GO annotation included phosphorylation (79 genes, 2.41%), integral membrane component (610 genes, 18.58%), and ATP binding (262 genes, 7.98%) (Figure 5).

**Figure 6:**
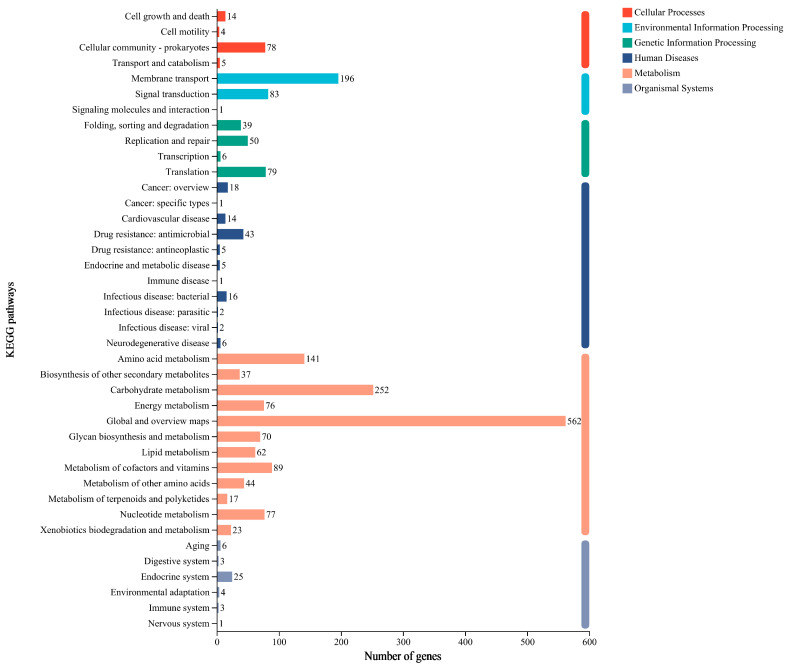
KEGG function annotation statistics for *Lp. plantarum* PCZ4.

To investigate the functional allocation within the metabolic pathways and cellular processes, KEGG annotation revealed that the PCZ4 genes were primarily enriched in the global and overview maps (562 genes, 31.81%) and carbohydrate metabolism (252 genes, 14.26%) within the metabolism category. Additionally, the membrane transport genes (196, 11.09%) were enriched in the environmental information processing category (Figure 6). The genes involved in the biosynthesis of secondary metabolites and the metabolism of terpenoids and polyketides were annotated in five and ten genes, respectively. Terpenes and polyketides are essential secondary metabolites of microorganisms with potential medicinal applications.

#### 3.2.3. Secondary Metabolite Analysis

**Figure 7:**
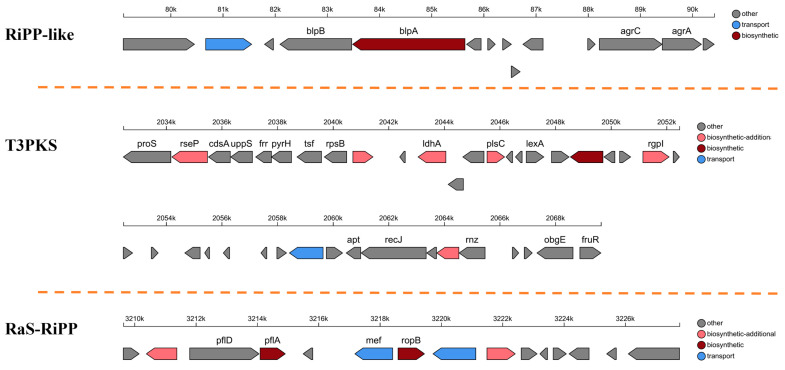
Gene cluster analysis of the secondary metabolite synthesis of *Lp. plantarum* PCZ4.

Three gene clusters involved in secondary metabolite production were identified using AntiSMASH 7.0. The gene clusters encode ribosome-synthesized post-translationally modified peptides (RiPP-like), type III polyketide synthases (T3PKS), and RaS-RiPP (Figure 7). The lengths of the three gene clusters in the PCZ4 whole genome were 12,155 bp, 41,171 bp, and 25,361 bp, respectively. RiPPs represent a diverse superfamily of natural products with immense potential for drug development [28]. T3PKS catalyzes the formation of various natural polyketide products with remarkable structural diversity and biological activities [29]. RaS-RiPP is code for ribosomally synthesized and post-translationally modified peptide (RiPP) natural products and is tailored by radical S-adenosylmethionine (RaS) enzymes [30]. PCZ4 can generate three gene clusters for secondary metabolite synthesis, demonstrating its potential in the discovery of natural medicinal products.

#### 3.2.4. Bacteriocin Production

Using BAGEL v4.0 software, two types of bacteriocins—pediocin and plantaricin_S—were predicted. The similarity between the Chromome.0.AOI-01 core protein and pedocin was 79.2%. Pediocin is a type of class IIa bacteriocin that effectively inhibits *L. monocytogenes*. The Chromome.0AOI-02 core protein displayed 100% similarity with plantaricin_S, a type of class IIb bacteriocin that consists of alpha and beta dipeptides.

**Figure 8:**
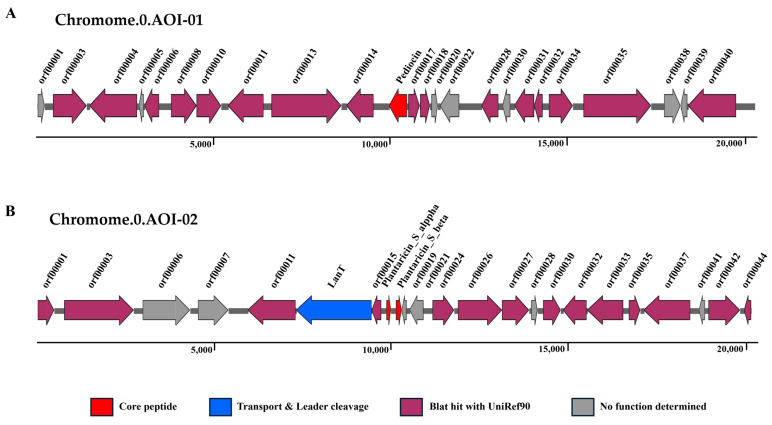
Bacteriocin cluster in the *Lp. plantarum* PCZ4 genome. (**A**) Pediocin. (**B**) Plantaricin_S. The arrows indicate the transcription direction of each gene.

Figure 8 shows the gene clusters involved in bacteriocin synthesis in PCZ4. In Chromome.0.AOI-01, *ped*A encodes the core protein; orf00004 encodes the MFS-type transporter; and orf00006 encodes the transcription regulatory factor AdhR. The operon was followed by ORFs encoding DNA-binding stress protein (orf00031), transcription regulator flp (orf00034), and neutral endopeptidase (orf00035) (Figure 8A).

In Chromosome.0.AOI-02, two genes, designated *pls*A and *pls*B, encode the alpha and beta peptides of plantaricin_S, respectively. Additionally, several other potential ORFs were identified, including those that may be responsible for regulating bacteriocin production. orf00011 encoded the bacteriocin secretion protein MesE; orf00012 encoded the transport/processing ATP-binding protein MesD; orf00015 encoded the transcriptional regulatory factor YqaE; orf00026 encoded a bacteriocin production-related histidine kinase; orf00027 encoded the response regulatory protein; and orf00037 encoded the multidrug output protein MepA (Figure 8B).

Additionally, new antibacterial peptides were predicted based on the structure of the antibacterial proteins. First, 15,838 ORFs were identified using the getORF function of EMBOSS software, with 12,600 located on chromosomes and 3238 on plasmids. Further signal peptide prediction using SignalP-6.0 revealed 33 ORFs with signal peptide sequences. After combining the prediction with CAMP and AntiBP2, two peptide sequences with antibacterial activity were identified: Chromome_1109 [94,929–95,039 bp] and Chromome_2485 [208,986–209,180 bp]. The two peptides did not have homologous sequences in the NCBI and AMP databases, indicating that they were novel bacteriocins. The two peptides were named Bac1109 and Bac2485, respectively, and their physicochemical properties are presented in Table 4. The amino acid sequence of Bac1109 is QVVVVIAALNCCSQSCAAGETKKVLHSHSACNTTIIG, while that of Bac2485 is STTGRRGWLDRLGIVFSGIIMIRHAPCPALVMTGRCIGCAQLKCSRLACTCCKGPRIFIKARNLG. Bac1109 contained 37 amino acids with a molecular weight of 3.76 kDa, and Bac2485 contained 65 amino acids with a molecular weight of 7.05 kDa. Both bacteriocins had an instability index < 50, indicating good stability. The signal peptide types of Bac1109 and Bac2485 were Sec/SPII and Tat/SPI, respectively. Bac2485 exhibited better hydrophilicity than Bac1109. Secondary structure prediction showed that Bac1109 and Bac2485 were primarily composed of extended chains and random curls. In summary, Bac1109 and Bac2485 are small-molecule antibacterial peptides with a single chain.

### 3.3. Bio-Preservative Potential of PCZ4 Crude Extract in Snakehead

When the volume ratio of ethyl acetate to the fermentation supernatant was 1:1, the concentration of the extracted bacteriocin protein was the highest, with the best antibacterial effect against *E. coli* and *S. aureus*. Therefore, this ratio was selected for crude PCZ4 extraction. The MIC of PCZ4 crude extract against *A. hydrophila* was 1.2 mg/mL; this concentration was used in groups A2 and B2.

**Figure 9:**
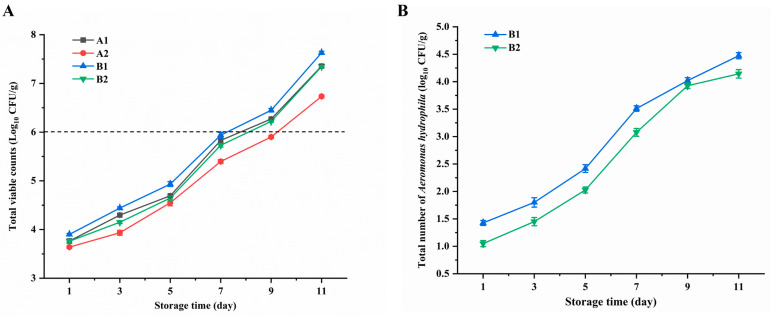
Changes in (**A**) total viable counts and (**B**) *Aeromonas hydrophila* of snakeheads treated at 4 °C. The results are expressed as the mean (*n* = 3) ± standard deviation.

The ability of PCZ4 crude extract to inhibit spoilage due to bacteria and foodborne pathogens (*A. hydrophila*) in snakehead was assessed. On day 1, the total bacterial counts for groups A1, A2, B1, and B2 were 3.8, 3.67, 3.9, and 3.79 log_10_ CFU/g, respectively (Figure 9A), with no significant differences observed between the groups (*p* > 0.05). With an increase in the number of refrigeration days, the total bacterial counts in the four groups increased gradually. The growth trend in group A2, which was treated with bacteriocin, was significantly slower than that in the other groups (*p* < 0.05). The standard limit for total bacterial counts in aquatic products is 6.0 log_10_ CFU/g. On day 7, the total bacterial counts in groups A1 (5.83 log_10_ CFU/g), B1 (5.93 log_10_ CFU/g), and B2 (5.73 log_10_ CFU/g) approached the upper limit, whereas on day 11, those in group A2 exceeded the limit. This shows that the addition of PCZ4 bacteriocins can effectively inhibit the growth of microorganisms and extend the shelf life of snakeheads by 2 days.

Additionally, the *A. hydrophila* cell count in group B1 was significantly higher (*p* < 0.05) than that in group B2, indicating that PCZ4 bacteriocins partially killed *A. hydrophila*. With the extension of the refrigeration time, the total *A. hydrophila* counts in both groups continued to increase. Conversely, the total counts in group B2 remained lower than those in group B1 (Figure 9B). This indicates that adding bacteriocins can, to some extent, inhibit the growth of *A. hydrophila* throughout the entire refrigeration process.

**Figure 10:**
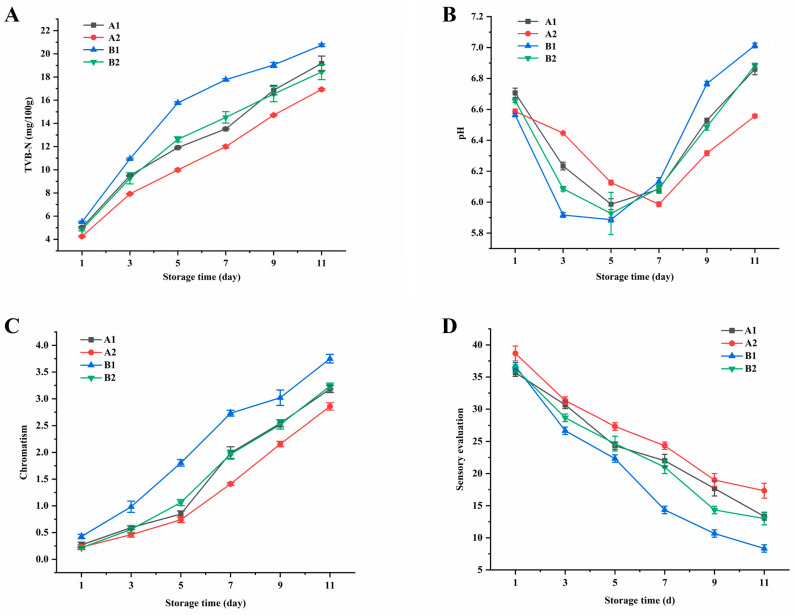
Changes in (**A**) TVB-N, (**B**) pH value, (**C**) chromatism, and (**D**) sensory evaluation of snakeheads with different treatments during storage at 4 °C. Results expressed as means (*n* = 3) ± standard deviation.

Figure 10A shows that all TVB-N values of the four groups were <6 mg/100 g on day 1, indicating that they were within the level I freshness range (≤15 mg/100 g, GB 2733-2015). As the storage time increased, only the B1 treatment group with *A. hydrophila* added exhibited a faster increase in TVB-N values, approaching the endpoint of level I freshness on day 5 and reaching the upper limit of level II freshness (≤20 mg/100 g) on day 11. On day 9, groups A1 and B2 reached the endpoint of level I freshness (16.9 mg/100 g; 16.5 mg/100 g, respectively) and close to the level II limit on day 11 (19.2 mg/100 g; 20.7 mg/100 g, respectively). Group A2, treated only with PCZ4 crude extract, maintained better freshness, reaching the upper limit of level I freshness on day 11 (16.9 mg/100 g). Using level I freshness as the standard, PCZ4 bacteriocin extended the shelf life of the snakehead by 2 days.

During storage, the pH values of all samples initially decreased and then subsequently increased (Figure 10B). The trend of pH change in treatment group A2 was slower than that in group A1. Similarly, the pH of group B2 increased more slowly than that of group B1. The pH of group B1 began to increase from day 3, while that of group A2 began to increase from day 7. By day 13, the pH values of A1, A2, B1, and B2 were 6.86, 6.55, 7.01, and 6.88, respectively, indicating that the bacteriocin treatment effectively delayed the pH increase.

With increasing storage time, the ΔE values of all four groups increased (Figure 10C). The ΔE value of treatment group B1 was significantly higher than that of the other treatment groups (*p* < 0.05). By day 11, the ΔE value of group B1 was >3.5, indicating a noticeable color change. The ΔE value of group A2 was significantly lower, and the ΔE value remained <3 during storage. Overall, these findings indicate that bacteriocin can delay the increase in the ΔE of snakehead samples.

The value of sensory evaluation of group B1 decreased faster, falling <20 on day 7. Groups A1 and B2 decreased <20 on day 9, whereas group A2 decreased <20 on day 11 (Figure 10D). This indicates that the PCZ4 crude extract can delay the spoilage of snakehead samples by inhibiting the growth and reproduction of microorganisms.

## 4. Discussion

Typically, screening bacteria with good antibacterial activity is a prerequisite for obtaining natural bacteriocins. For example, the CFS of *Lactobacillus paracasei* CNCM I-5369, isolated from a traditional Algerian dairy product, is active against a panel of pathogenic *E. coli* strains [31]. In our study, *Lp. pentosus* PCZ4, isolated from Sichuan pickles, exhibited broad-spectrum antibacterial activity. From the antibacterial spectrum findings, PCZ4 showed good antibacterial effects against various Gram-positive bacteria (including *S. aureus* and *B. subtilis*) and Gram-negative bacteria (including *E. coli*, *Salmonella* sp., and *P. aeruginosa*), and even against common pathogenic bacteria found in aquatic products, such as *Aeromonas*.

*Lp. pentosus*—commonly isolated from commercially fermented products—is a potent bacteriocin producer [32,33]. The stationary phase of PCZ4 growth exhibited maximum antibacterial activity. Similar results were observed in *Lp. Plantarum* [34], *Lp. plantarum* GZ1-27 [35], and *Lacticaseibacillus rhamnosus* XN2 [36]. Within 48 h, the antibacterial activity did not decrease, indicating that the antibacterial substances produced by PCZ4 have stable properties and do not easily degrade or lose their activity [37].

The genome size of *Lp. pentosus* is usually >3.6 Mb, e.g., *Lp. pentosus* CF2-10N (3.65 Mb), *Lp. pentosus* LPG1 (3.62 Mb), and *Lp. pentosus* 9D3 (3.65 Mb) strains [38,39,40]. The genome size of *Lp. pentosus* is larger than that of *Lp. plantarum* (usually >3.20 Mb) and other *Lactobacillus* spp., such as *Lactobacillus rhamnosus* DSM 14870 (3.01 Mb), *Lactobacillus casei* LC5 (3.13 Mb), *Lactobacillus fermentum* F-6 (2.06 Mb), and *Lactobacillus acidophilus* NCFM (1.99 Mb) [41]. This may be attributed to the ecological flexibility and diversity of the ecological niches of *Lp. pentosus* [42]. A larger genome is obtained through horizontal gene transfer via mobile elements such as plasmids, transposons, prophages, and integrons [43,44]. *Lp. pentosus* PCZ4 (3.70 Mb) contains one prophage, two insertions, four gene islands, four transposons, and 12 CRISPR loci, enriching the genome to a certain extent.

Bacteriocin mining leverages a wealth of data from bacterial genome or metagenomic sequencing projects, with a focus on the characteristics of bacteriocin gene clusters, particularly the modification genes that dictate bacteriocin diversity. BAGEL4.0 identified the presence of two bacteriocins in PCZ4: pediocin (Class IIa) and plantaricin_S (Class IIb).

Pediocins are characterized as small (<5 kDa) unmodified cationic peptides consisting of a conserved hydrophilic N-terminal region. This contains the YGNGV motif and a variable C-terminal portion, e.g., AcH, JD, and pediocin PA-1 [45]. The pediocin PA-1 gene cluster comprises an operon containing four genes: *ped*A, *ped*B, *ped*C, and *ped*D. However, reports have shown that individual expression of *ped*A is sufficient to demonstrate the activity of pediocin PA-1 [46]. These natural bacteriocins exert potent antimicrobial activity against Gram-positive bacteria, particularly against *L. monocytogenes* [47]. This makes them suitable for various food product applications to control the growth of this pathogen. This can be achieved either by adding pediocin-producing strains or by optimizing the concentration of pediocin in the food matrix [3]. Furthermore, pediocin is effective against Gram-negative microorganisms [48,49].

Plantaricin_S—a two-peptide class IIb bacteriocin—was previously identified in *Lp. plantarum* LPCO10 isolated from fermented green olives [50]. Its antibacterial activity depends on the complementary action of alpha and beta peptides [51,52]. The helix−helix alignment of the two peptides and the interaction between the conserved GxxxG/AxxxA motifs are crucial for their antimicrobial activity. PCZ4 possesses genes that synthesize both alpha and beta peptide chains. Additionally, Rekha Kumari et al. [53] confirm the presence of plantaricin S in the human gut.

Researchers have also explored potential antibacterial peptides using sequence analysis and prediction from scratch based on ORFs. Because bacteriocins are typically short peptides with signal peptide sequences, signal peptide prediction software combined with antimicrobial peptide prediction tools can identify ORFs encoding potential bacteriocins secreted extracellularly. Meng et al. [54] demonstrated this process by isolating the gene *pln*1 from 18,575 ORFs in the *Lp. plantarum* 163 genome. After cloning and expression, Pln1 exhibited superior antimicrobial activity against methicillin-resistant *Staphylococcus epidermidis* compared to that of the well-established bacteriocin nisin. Employing this method, we predicted two potential class IId bacteriocins, Bac1109 and Bac2485, in the PCZ4 genome sequence. However, the bacteriocins predicted by this approach require experimental validation to confirm their activity.

Biopreservation applications have broad potential for storing aquatic products, such as crucian carp, large yellow croaker, seabass, and obscure pufferfish [55,56,57]. The preservative effect of PCZ4 crude extract on snakehead quality during storage was evaluated using combined sensory, microbiological, and physicochemical analyses. PCZ4 can not only inhibit the growth of the total bacterial count but also suppress the growth of the Gram-negative foodborne pathogen *A. hydrophila*. *A. hydrophila* is an important waterborne pathogen that can contaminate various aquatic products [58]. Several LAB strains, including *Lactobacillus rhamnosus*, *Lactobacillus casei,* and *Lp. plantarum*, exhibit antagonistic activity against *A. hydrophila* [59]. Because microbial growth is closely related to aquatic product spoilage, inhibiting microbial growth can prolong shelf life. Various bacteriocins exert inhibitory effects against foodborne spoilage and pathogenic bacteria. For example, *Lacticaseibacillus paracasei* MG847589 and bacteriocin inhibit *S. aureus* from 6.52–2.10 log_10_ CFU/g during soft white cheese storage [60]. After treatment with PCZ4 crude extract, the total bacterial count in snakeheads decreased from 7.36 to 6.73 log_10_ CFU/g at day 11, indicating a significant antibacterial effect during snakehead storage.

Additionally, PCZ4 delayed the increase in TVB-N and pH throughout snakehead storage. The accumulation of TVB-N during food spoilage is primarily caused by protein decomposition by microorganisms and enzymes that produce trimethylamine, dimethylamine, ammonia, and other volatile basic nitrogenous compounds associated with seafood spoilage [61]. The changes in pH during seafood spoilage are complex, and LAB fermentation and glycolysis reactions produce lactic acid. This process degrades ATP and accumulates phosphoric acid, leading to a decrease in pH, whereas protein decomposition produces alkaline products, thereby increasing the pH. The increase in TVB-N and pH after treatment was significantly delayed compared to that of the control group. This indicates that the PCZ4 crude extract reduces protein degradation by inhibiting microbial growth, thereby reducing the formation of alkaline nitrogen-containing compounds. The potential of the bacteriocin for controlling the increase in TVB-N and pH in snakeheads is consistent with the observations made using bacteriocin GP1 in fish fillets. Combining the total bacterial count and the TVB-N result, PCZ4 bacteriocin can extend the shelf life of snakeheads by 2 days.

## 5. Conclusions

The LAB strain *Lp. pentosus* PCZ4—a strain with broad-spectrum antibacterial activity—was isolated from Sichuan pickles. By combining WGS and bioinformatics analyses, two bacteriocins (pedocin and plantaricin_S) were identified, and two new antimicrobial peptides (Bac1109 and Bac2485) were predicted from scratch. Furthermore, the PCZ4 crude extract effectively prolonged the refrigerated shelf life of snakeheads. This indicates that *Lp. pentosus* PCZ4 is a highly active bacterial strain that produces various antibacterial substances, and that it can be used as a food preservative. Further studies will be conducted to determine the antibacterial activity and mode of action of the four types of PCZ4 bacteriocins after heterologous expression.

## Figures and Tables

**Table 1:** Antimicrobial activity of ten LAB-like morphological strains.

Strains	Diameter of Inhibition Zone (cm)									
	Gram-Positive Bacteria				Gram-Negative Bacteria					
	*Bacillus subtilis* ATCC 6633	*Staphylococcus aureus* PS 011	*Staphylococcus aureus* ATCC BAA 1717	*Staphylococcus aureus* subsp. ATCC 6538	*Escherichia coli* CMCC 44102	*Escherichia coli* PS 075	*Salmonella* sp. PS 105	*Salmonella* sp. FA 108	*Pseudomonas aeruginosa* FA203	*Aeromonas veronii* JS 008
PCZ4	1.23 ± 0.03 ab	1.75 ± 0.04 ab	1.23 ± 0.10 ab	1.22 ± 0.06 a	1.28 ± 0.06 a	1.13 ± 0.06 ab	1.25 ± 0.10 a	1.12 ± 0.03 ab	1.40 ± 0.07 a	1.25 ± 0.05 a
PZ1	1.25 ± 0.05 a	1.80 ± 0.14 a	1.23 ± 0.03 ab	1.17 ± 0.03 a	1.23 ± 0.06 ab	1.13 ± 0.03 ab	1.22 ± 0.06 a	1.15 ± 0.05 a	1.28 ± 0.04 a	1.17 ± 0.06 ab
L1	1.17 ± 0.03 abc	1.75 ± 0.07 ab	1.20 ± 0.05 abc	1.13 ± 0.06 a	1.18 ± 0.06 abc	1.03 ± 0.03 bc	1.18 ± 0.03 a	1.03 ± 0.03 b	1.23 ± 0.04 b	1.12 ± 0.10 b
P27	1.18 ± 0.08 abc	1.73 ± 0.04 ab	1.13 ± 0.03 bcd	1.12 ± 0.10 b	1.17 ± 0.08 bc	1.05 ± 0.09 bc	1.20 ± 0.05 a	1.03 ± 0.06 b	1.10 ± 0.14 bc	1.20 ± 0.05 ab
P23B	1.13 ± 0.06 c	1.63 ± 0.04 c	1.22 ± 0.06 ab	1.07 ± 0.12 b	1.17 ± 0.06 bc	1.07 ± 0.08 bc	1.12 ± 0.03 a	1.07 ± 0.08 ab	1.50 ± 0.14 a	1.13 ± 0.06 ab
YC3B	1.13 ± 0.03 c	1.50 ± 0.07 cd	1.27 ± 0.06 ab	1.12 ± 0.03 b	1.18 ± 0.03 abc	1.02 ± 0.03 bc	1.22 ± 0.06 a	1.07 ± 0.06 ab	1.53 ± 0.04 a	1.08 ± 0.08 b
HPZ2A	1.15 ± 0.05 bc	1.40 ± 0.00 d	1.17 ± 0.03 ac	1.13 ± 0.06 ab	1.17 ± 0.06 bc	1.02 ± 0.03 c	1.18 ± 0.08 a	1.10 ± 0.09 ab	1.38 ± 0.04 a	1.18 ± 0.08 ab
HPZ2B	1.17 ± 0.03 abc	1.55 ± 0.07 cd	1.05 ± 0.05 bd	1.13 ± 0.06 ab	1.12 ± 0.03 c	1.07 ± 0.06 bc	1.13 ± 0.10 a	1.03 ± 0.06 b	1.05 ± 0.07 c	1.10 ± 0.05 b
HPZ2C	1.13 ± 0.06 c	1.58 ± 0.11 cd	1.12 ± 0.03 cd	1.13 ± 0.06 ab	1.12 ± 0.03 c	1.20 ± 0.05 a	1.18 ± 0.06	1.07 ± 0.06 ab	1.45 ± 0.07 a	1.15 ± 0.05 ab
YY16	1.18 ± 0.03 abc	1.45 ± 0.07 d	1.05 ± 0.05 cd	1.10 ± 0.10 b	1.17 ± 0.06 bc	1.13 ± 0.03 ab	1.17 ± 0.06	1.08 ± 0.03 ab	1.48 ± 0.04 a	1.15 ± 0.05 ab

Lowercase letters (a, b, c, and d) within each column indicate significant differences according to Duncan’s multiple range test (*p* < 0.05).

**Table 2:** Survival of ten LAB-like morphological strains after exposure to simulated gastric juice and bile salt pancreatic conditions.

Strains	Survival Rate in Simulated Gastric Juice (%)	Growth Rate with Bile Salt (%)	
		0.20%	0.30%
PCZ4	110.72 ± 5.39 d	66.7 ± 0.04 b	63.6 ± 0.64 b
PZ1	48.33 ± 1.66 h	66.8 ± 0.02 b	65.5 ± 0.65 a
L1	57.52 ± 4.82 g	75.8 ± 0.05 a	60.8 ± 0.11 c
P27	133.50 ± 3.87 c	16.4 ± 0.06 i	8.9 ± 0.11 i
P23B	138.24 ± 2.66 bc	65.8 ± 0.07 c	49.3 ± 0.49 f
YC3B	111.23 ± 7.95 d	61.4 ± 0.07 e	51.3 ± 0.12 e
HPZ2A	160.47 ± 4.06 a	51.0 ± 0.04 f	44.0 ± 0.02 g
HPZ2B	76.01 ± 7.32 f	61.7 ± 0.10 d	54.1 ± 0.12 d
HPZ2C	95.14 ± 21.52 e	40.7 ± 0.20 g	39.1 ± 0.27 g
YY16	144.00 ± 4.00 b	28.7 ± 0.20 h	25.4 ± 0.28 h

Lowercase letters (a to i) within each column indicate significant differences according to Duncan’s multiple range test (*p* < 0.05).

**Table 3:** Overview of the WGS analysis results of *Lp. plantarum* PCZ4.

Features	
**Genomic assembly and prediction**	
Chromosome No.	1
Plasmid No.	3
Genome size (bp)	3,702,061
GC content (%)	46.19
CDS	3283
rRNA No.	16
tRNA No.	67
sRNA No.	47
**Functional database annotations**	
NR No.	3266
Swiss-Prot No.	2306
Pfam No.	2682
COG No.	2575
CO No.	2031
KEGG No.	1767
**Mobile Genetic elements**	
Gene island No.	4
Prophage No.	1
CRISPR No.	12
Integron No.	0
Insertion sequence No.	2
Transposon No.	4

**Table 4:** Amino acid sequence characteristics of Bac1109 and Bac2485.

Name	Amino Acid Number	Molecular Weight/kDa	Theoretical Isoelectric Point (pI)	Positively Charged Amino Acid (Arg + Lys)	Negatively Charged Amino Acid (Asp + Glu)	Instability Index	Hydrophilicity Index	Antimicrobial Peptides Possibility	Signal Peptide Type
Bac1109	37	3.76	7.9	2	1	38.05	0.63	0.87	Sec/SPII
Bac2485	65	7.05	10.37	11	1	35.27	0.355	0.82	Tat/SPI

## Data Availability

The original contributions presented in the study are included in the article; further inquiries can be directed to the corresponding author.
